# A Multi-Target Approach toward the Development of Novel Candidates for Antidermatophytic Activity: Ultrastructural Evidence on α-Bisabolol-Treated *Microsporum gypseum*

**DOI:** 10.3390/molecules200711765

**Published:** 2015-06-26

**Authors:** Carlo Romagnoli, Anna Baldisserotto, Gemma Malisardi, Chiara B. Vicentini, Donatella Mares, Elisa Andreotti, Silvia Vertuani, Stefano Manfredini

**Affiliations:** 1 Department of Life Sciences, University of Modena and Reggio Emilia, viale Caduti in Guerra 127, 41121 Modena, Italy; E-Mails: carlo.romagnoli@unimore.it (C.R.); elisa.andreotti@unimore.it (E.A.); 2 Department of Life Sciences and Biotechnology, Master Course in Cosmetic Science and Technology, University of Ferrara, via Fossato di Mortara 17-19, 44121 Ferrara, Italy; E-Mails: bldnna@unife.it (A.B.); gemma.malisardi@unife.it (G.M.); chiara.vicentini@unife.it (C.B.V.); donatella.mares@unife.it (D.M.); silvia.vertuani@unife.it (S.V.)

**Keywords:** antifungal activity, dermatophytes, *M. gypseum*, α-bisabolol, TEM, skin lightener

## Abstract

Multi-target strategies are directed toward targets that are unrelated (or distantly related) and can create opportunities to address different pathologies. The antidermatophytic activities of nine natural skin lighteners: α-bisabolol, kojic acid, β-arbutin, azelaic acid, hydroquinone, nicotinamide, glycine, glutathione and ascorbyl tetraisopalmitate, were evaluated, in comparison with the known antifungal drug fluconazole, on nine dermatophytes responsible for the most common dermatomycoses: *Microsporum gypseum*, *Microsporum canis*, *Trichophyton violaceum*, *Nannizzia cajetani*, *Trichophyton mentagrophytes*, *Epidermophyton floccosum*, *Arthroderma gypseum*, *Trichophyton rubrum* and *Trichophyton* *tonsurans*. α-Bisabolol showed the best antifungal activity against all fungi and in particular; against *M. gypseum*. Further investigations were conducted on this fungus to evaluate the inhibition of spore germination and morphological changes induced by α-bisabolol by TEM.

## 1. Introduction

The skin is our first line of defense against the outside world. As the body’s border, it is subject to various microbiological attacks. In particular, approximately 25% of the world’s population suffers from skin mycosis [1], which represents one of the main causes of morbidity and mortality [2] and the seriousness of the disease is steadily increasing. Dermatophytoses are very fastidious and difficult to manage, and their therapy is complex and associated with high costs. Our research group has long been involved in the search for new molecules with antifungal activity, exploring not only the fields of unknown natural [3,4,5,6] and synthetic [7,8,9,10] substances but also those substances already used in other clinical fields. Our main interest is in the discovery of molecules with multiple activities (multi-target-drugs), the advantages from such an approach include not only a better balance between activity and side effects but also a reduction in cost and time to develop and reach the market. If multi-target strategies are directed toward targets that are unrelated (or distantly related) an opportunity maybe created to target different pathologies. In these regards, it is well known that skin hyperpigmentation is a relevant problem, affects a large portion of the world’s population, and is a particular social issue for black and elderly people. This context has prompted the search for more effective treatments in dermatology and this problem is attributed to the over-accumulation of melanin in skin cells. Several molecules, mostly of natural origin, have been used over the last few years to counteract this effect. While the mechanisms of action are different, these molecules reduce melanin content. In a completely different field, melanin biosynthesis inhibitors have long been used for the control of plant pathogenic fungi [9,11,12]. We began an explorative investigation of these known melanin inhibitors, which may be used as antifungal agents in combination with known or investigational therapeutics. Agents that are typically used for dermatological purposes (e.g., skin lighteners), whose low toxicity, pharmacokinetics and pharmacodynamics are already known, will accelerate the process of developing innovative, less toxic and safer drug candidates. In particular, α-bisabolol, kojic acid, β-arbutin, azelaic acid, hydroquinone, nicotinamide, glycine, glutathione and ascorbyl tetraisopalmitate were selected. These nine molecules are already known in the literature for their action as skin lighteners [13,14,15,16,17] and are present in several commercial products.

In this work, the antidermatophytic activity of these substances was tested on nine dermatophytes responsible for the most common dermatomycoses: *Microsporum gypseum*, *Microsporum canis*, *Trichophyton violaceum*, *Nannizzia cajetani*, *Trichophyton mentagrophytes*, *Epidermophyton floccosum*, *Arthroderma gypseum*, *Trichophyton rubrum* and *Trichophyton tonsurans*.

**Figure 1:**
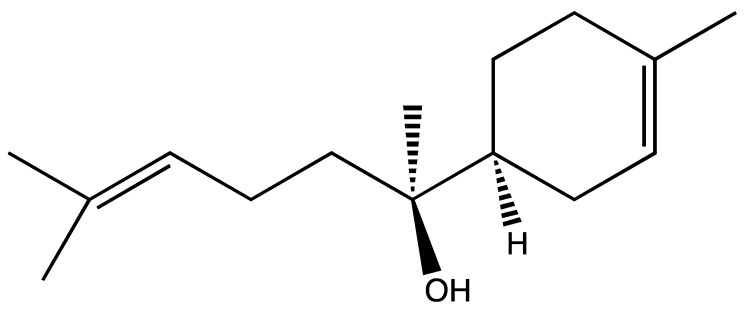
Chemical structure of α-bisabolol.

After a preliminary investigation, α-bisabolol emerged as the most active compound; thus, the following experiments focused on this molecule and its effects on the most sensitive of the nine fungi, *M. gypseum*. This molecule (Figure 1) is a sesquiterpene alcohol found in the essential oils of several plants, such as *Arnica longifolia*, *Aster esperius*, *Chrysothamnus nauseosus* [18], and *Chamomilla* sp. [19], and its antibacterial and antifungal activities are well-known in the literature [20]. In particular, investigations were conducted by TEM to confirm whether the mechanism of action for the inhibition of ergosterol synthesis by α-bisabolol, as suggested by Pauli [19] for other fungi, was also responsible for its antifungal activity [21].

## 2. Results and Discussion

### 2.1. Antifungal Activity

The growth inhibition data are shown in Table 1.

**Table 1:** Percent growth inhibition of dermatophytes treated with nine skin lighteners at 100 or 200 μg/mL. Each value is the mean of three measurements.

		Kojic Acid	β-Arbutin	Azelaic Acid	Hydroquinone	Nicotinamide	α-Bisabolol	Glycine	Glutathione	Ascorbyl TIP
***M. gypseum***	100	3.5 ± 0.1	7.9 ± 0.2	6.9 ± 0.2	17.1 ± 0.1	9.8 ± 0.1	79.8 ± 0.4	+	+	16.6 ± 0.3
	200	7.5 ± 0.2	27.9 ± 0.1	19.8 ± 0.1	51.2 ± 0.1	18.3 ± 0.4	83.7 ± 0.2	0.8 ± 0.1	+	21.3 ± 0.2
***M. canis***	100	22.6 ± 0.3	−11.8 ± 0.2	−5.9 ± 0.1	20.0 ± 0.1	2.4 ± 0.1	34.7 ± 0.2	0.0	0.0	+
	200	29.0 ± 0.1	−2.9 ± 0.2	5.9 ± 0.3	46.7 ± 0.2	12.0 ± 0.1	43.1 ± 0.2	+	3.8 ± 0.3	+
***T. violaceum***	100	18.2 ± 0.2	−2.8 ± 1.4	−8.2 ± 0.9	20.0 ± 1.2	3.4 ± 1.8	31.0 ± 0.8	+	9.8 ± 2.3	+
	200	19. 9 ± 1.1	−5.0 ± 0.7	2.5 ± 1.6	30.6 ± 1.3	+	38.0 ± 1.8	0.0	12.0 ± 1.4	15.6 ± 2.0
***N. cajetani***	100	2.7 ± 1.1	6.9 ± 1.6	0.9 ± 1.9	0.0	14.4 ± 0.7	60.1 ± 1.3	0.0	+	+
	200	8.0 ± 1.0	7.6 ± 1.4	0.0	4.2 ± 0.3	25.1 ± 0.6	62.7 ± 1.5	+	5.2 ± 1.3	0.0
***T. mentagroph***	100	0.0	−1.5 ± 0.5	0.0	29.7 ± 1.8	50.8 ± 1.8	67.0 ± 1.3	6.1 ± 0.6	+	9.8 ± 1.1
	200	6.3 ± 1.2	0.0	9.3 ± 0.8	59.4 ± 1.4	78.0 ± 3.1	70.9 ± 1.7	+	+	19.6 ± 0.8
***E. floccosum***	100	1.9 ± 1.7	−1.6 ± 0.5	0.3 ± 0.1	2.5 ± 0.2	7.8 ± 0.7	31.8 ± 1.1	+	0.6 ± 0.2	+
	200	2.5 ± 0.9	−1.3 ± 0.5	3.9 ± 1.0	5.4 ± 0.8	+	33.1 ± 1.9	+	0.0	6.4 ± 0.9
***A. gypseum***	100	10.0 ± 1.2	0.0	7.4 ± 1.4	−11.0 ± 0.9	1.9 ± 1.2	62.0 ± 1.7	+	0.6 ± 2.1	0.7 ± 0.5
	200	11.3 ± 1.2	2.3 ± 1.8	3.7 ± 1.4	2.4 ± 1.1	7.1 ± 0.6	65.8 ± 1.3	+	1.3 ± 1.0	+
***T. rubrum***	100	−0.4 ± 0.8	6.1 ± 1.4	0.8 ± 0.5	−2.3 ± 1.9	4.5 ± 0.3	14.6 ± 1.9	+	2.6 ± 0.2	0.0
	200	2.9 ± 0.7	6.8 ± 1.3	1.6 ± 0.7	2.9 ± 0.6	10.8 ± 1.1	37.4 ± 1.4	+	+	+
***T. tonsurans***	100	0.0	4.4 ± 0.5	−2.6 ± 1.2	40.0 ± 1.1	9.3 ± 1.3	69.6 ± 1.9	+	+	+
	200	5.0 ± 0.5	11.1 ± 0.7	0.0	55.00 ± 1.5	+	71.7 ± 1.2	0.0 ± 0.8	+	+

In general, the nine substances showed low inhibition of growth. In fact, after treatment with β-arbutin, increased fungal growth was observed for *M. canis* and *T. violaceum*. The only substances that showed significant inhibition values were hydroquinone and α-bisabolol. The former showed inhibition values greater than 50% at the highest dose (200 μg/mL) for *M. gypseum* (51%), *T. tonsurans* (55%) and *T. mentagrophytes* (59.5%). At the lower dose (100 μg/mL), a 40% inhibition value was observed for *T. tonsurans*. When treated with 100 μg/mL α-bisabolol, at least 30% inhibition was observed for all fungi, with the exception of *T. rubrum*. For both the lower and higher doses of α-bisabolol, inhibition was greater than 50% for five fungi (*M. gypseum*, *N. cajetani*, *T. mentagrophytes*, *T. tonsurans* and *A. gypseum*). In particular, *T. mentagrophytes* and *T. tonsurans* were sensitive to treatment, reaching approximately 70% inhibition for both dose levels. For treatments with 100 μg/mL and 200 μg/mL of α-bisabolol, the highest inhibition levels were observed in *M. gypseum* at 79.8% and 83.7%, respectively. Due to this high inhibition activity, we chose *M. gypseum* for further studies at the ultrastructural level to determine morphological changes induced by α-bisabolol.

In Table 2 are shown the IC_50_ values of α-bisabolol, in comparison with the well known antifungal agent, fluconazole.

**Table 2:** IC_50_ values of α-bisabolol and Fluconazole on dermatophytes. Each value is the mean of three measurements.

	α-Bisabolol	Fluconazole
	IC_50_ μg/mL	
***M. gypseum***	35.24 ± 0.9	18.5 ± 0.2
***M. canis***	>200	29.6 ± 0.3
***T. violaceum***	>200	31.03 ± 0.9
***N. cajetani***	85 ± 0.6	>200
***T. mentagrophytes***	60.51 ± 0.3	3.53 ± 0.8
***E. floccosum***	>200	0.08 ± 0.6
***A. gypseum***	81.69 ± 0.4	>200
***T. rubrum***	>200	37.16 ± 0.6
***T. tonsurans***	49.38 ± 0.7	19.41 ± 0.5

IC_50_ values confirmed the interesting activity of α-bisabolol, in particular on *M. gypseum* (35.24 μg/mL) and *T. tonsurans* (49, 38 μg/mL), it is remarkable that on two fungi (*N. cajetani* and *A. gypseum*) the IC_50_ was even lower than that of fluconazole.

### 2.2. Effect of Time and Spore Density on Percent Reduction of Resazurin

**Figure 2:**
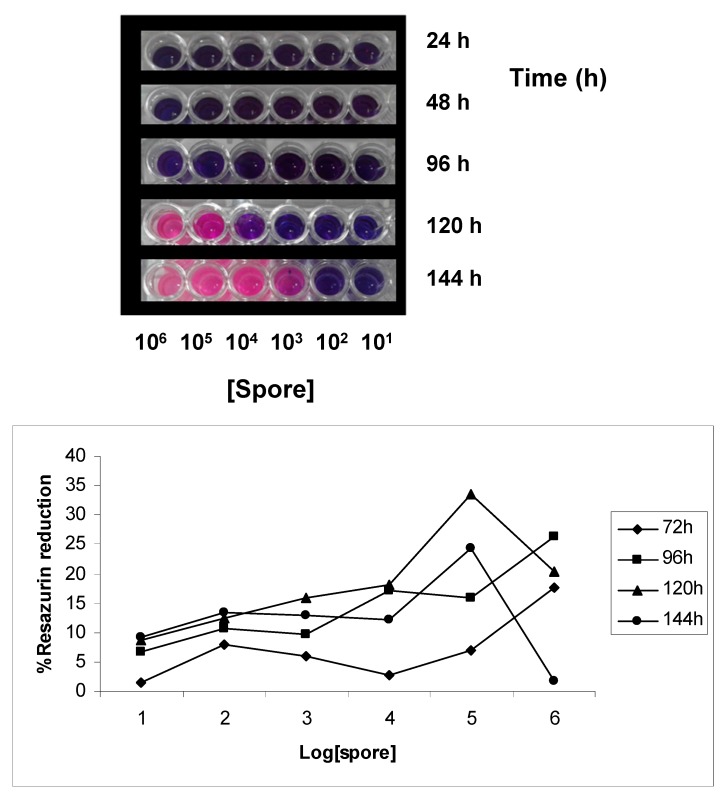
Effect of time and spore density on percent reduction of Resazurin. The highest reduction occurred after 120 h of incubation with a spore density of 10^5^ spores.

The effect of spore density and incubation time on reduction of Resazurin, are illustrated in Figure 2. The blue color indicates absence of cell viability while the pink color indicates the presence of cell viability. It can be seen that the first signs of cell viability appeared at a density of 10^5^ spores/mL and after an incubation time at 24 °C of 120 h. We considered these values of spore density and incubation time the best for the evaluation of inhibition of spore germination.

### 2.3. Inhibition of Spore Germination by Resazurin Assay

Table 3 presents the values of percent inhibition rate of spore germination with α-bisabolol at the concentrations of 100 and 200 μg/mL. It appeared to be a good inhibitor of spore germination at both tested doses of 100 and 200 μg/mL (52.08% and 59.32% inhibition respectively). It is noteworthy that these small differences in inhibition of spore germination corresponded well with the effect of α-bisabolol on growth at both doses (79.8% and 83.7% inhibition respectively).

**Table 3:** Percent inhibition values on spore germination.

α-Bisabolol Concentration	%Inhibition of Spore Germination
100 μg/mL	52.08 ± 0.9
200 μg/mL	59.32 ± 1.3

### 2.4. TEM

**Figure 3:**
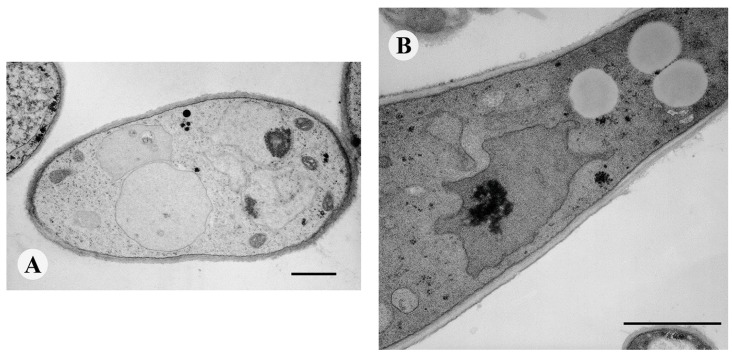
(**A**) Untreated control hypha of *M. gypseum* observed by TEM. The nucleus and the cell wall are normally structured; (**B**) TEM of *M. gypseum* treated with 100 μg/mL α-bisabolol. The nucleus shows deep invaginations. Bar, 1 μm.

When examined by TEM, the mycelium of the *M. gypseum* control sample appeared normal: all organelles, such as the nucleus and mitochondria, were normally shaped, and the plasma membrane was unfolded with a uniform appearance (Figure 3A).

After 24 h of treatment at the lower α-bisabolol concentration of 100 µg/mL, morphological changes were observed. As shown in Figure 3B, the nuclei are irregularly shaped, presenting numerous anomalous lobes and invaginations; even the nucleoli appear frayed and not very compact.

**Figure 4:**
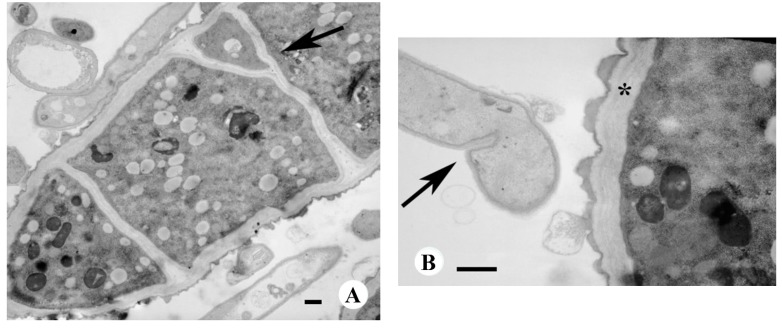
TEM of *M. gypseum* treated with 200 μg/mL α-bisabolol. (**A**) Septa are in closer proximity with each other and have anomalous shapes; arrow indicates a bifidous septum; (**B**) Arrow indicates the early formation of a septum in the sub apical area; asterisk indicates an abnormal multilayer cell wall. Bar, 1 μm.

In Figure 4A, after treatment at the higher 200 μg/mL concentration, the outer cell wall presents significant abnormalities. In particular, the anomalous formation of numerous and recurring septa are visible; the septa often have altered shapes, such as the bifid septum indicated by the arrow. In addition, the septa are located in unusual areas. In Figure 4B, there is evidence of the early formation of a septum in the sub apical area, which is a location where parietal components are still in phase of linkage. In the same figure, a thick cell wall (indicated by an asterisk) is present in an adjacent cell, made of many layers and it is also visible an abnormal extrusion of cell wall materials from the outer layer.

Finally, increased vacuolization is observed within the cytoplasm after treatment with 200 μg/mL of α-bisabolol.

Our study shows that, among the nine skin lighteners tested as antifungal agents, α-bisabolol showed promising properties, due to its ability to inhibit the growth of all tested dermatophytes, especially *M. gypseum*. The IC_50_ of α-bisabolol on *M. gypseum* suggested us to choose this fungus to better understand the antidermatophytic activity. The first step was the evaluation of spore germination inhibition with the Resazurin assay. This is a widespread test in agronomy, but only little applied on dermatophytes and never used on *M. gypseum*. So it was necessary to adapt the procedure, by determing the right number of spore and the time of germination of this fungus. The results obtained with Resazurin assay on spore germination, confirmed the good inhibition activity of α-bisabolol on *M. gypseum*.

TEM observations were performed on an α-bisabolol-treated *M. gypseum* sample to better understand the mechanism of action of this anti-fungal agent. Upon treatment with α-bisabolol, several morphological abnormalities were observed in *M. gypseum*. At a low α-bisabolol concentration, the nuclear envelope presented numerous lobes, whereas at a higher dose, the aberrations are widespread, even affecting the cell wall structure. Both aspects are likely related to altered microtubule behavior. α-Bisabolol may affect the dynamic microtubules responsible for maintaining the nuclear shape, causing alterations in the nuclear envelope [22,23]. Additionally, it is also possible that α-bisabolol targets the microtubules responsible for directing cellular wall materials toward the cell periphery. It is known that MTs and other cytoskeleton components are responsible for the normal assembly of parietal components and are involved in the transport and release of vesicles containing the construction materials [24]. Disruption of the MTs leads to altered vesicle distributions [25] and thus results in the irregular build-up of wall materials to apical locations along the cell wall. This proposed mechanism could also explain the anomalous septa appearances in the apical zone and the increased concentration of septa. The observed effects correspond with the effects of α-bisabolol when used as a biosynthetic inhibitor of ergosterol, a sterol specifically located in the membrane of fungi, as suggested by Pauli (2006). In this previous study, by comparing the chemical structures of α-bisabolol and zymosterol, Pauli suggested treatment with α-bisabolol prevented the formation of fecosterol from zymosterol, which is the first fungi-specific step in ergosterol biosynthesis. Thus, α-bisabolol has a mechanism of action common to several antifungal drugs [26,27].

α-Bisabolol may exists as four different isomers. The natural, pure isomer is the RR (−) enantiomer, which can only be extracted from natural origins. While the (−) and (+) racemic mixtures of α-bisabolol can be chemically synthesized in the laboratory, the separation of the two enantiomers is economically disadvantageous. The racemic mixtures have half the effective biological activity of the pure, naturally derived (−)α-bisabolol.

## 3. Experimental Section

### 3.1. Chemicals

The tested substances (α-bisabolol, kojic acid, β-arbutin, azelaic acid, hydroquinone, nicotinamide, glycine, glutathione and ascorbyl tetraisopalmitate) were purchased from Fagron, API Corporation, Res Pharma, Sarfam Comercial Importadora Ltda. (Sao Paolo, SP, Brazil) and Nikko Chemicals Co., Ltd. (Tokyo, Japan).

Fungal culture medium was purchased from Difco Laboratories, Inc. (Becton, Dickinson and Company, Cockeysville, MD, USA).

The chemicals used for fixations and solvents were purchased from Sigma-Aldrich SRL, Milano, Italy.

### 3.2. Microorganisms

Seven of the specific fungi strains investigated in this study were purchased from the Centraal Bureau voor Schimmelcultures (CBS), Baarn, The Netherlands: *Nannizzia cajetani* Ajello, strain CBS 495.70; *Epidermophyton floccosum* (Hartz) Langeron and Milochevitch, strain CBS 358.93; *Trichophyton violaceum* Malmsten, strain CBS 459.61; *Trichophyton tonsurans* Malmsten, strain CBS 483.76, *Trichophyton mentagrophytes* (Robin) Blanchard, strain CBS 160.66; *Microsporum canis* Bodin, strain CBS 4727; and *Arthroderma gypseum* (Nann.) Weitzman, McGinnis, A.A. Padhye and Ajello, strain CBS 286.63. The remaining two strains were purchased from the Institute of Hygiene and Epidemiology-Mycology Laboratory (IHME), Brussels, Belgium: *Trichophyton rubrum* (Castellani) Sabouraud, strain IHME 4321; *Microsporum gypseum* (Bodin) Guiart e Grigorakis, strain IHME 3999. All dermatophytes were maintained at 4 °C as agar slants on Sabouraud dextrose agar (SDA; Difco Laboratories, Inc.).

### 3.3. Antifungal Activity

Antifungal activity was determined as follows. Each test substance was dissolved in dimethylsulfoxide (DMSO) and aseptically mixed with sterile medium (SDA) at 45 °C to concentrations of 100 and 200 μg/mL. The DMSO concentration in the final solution was adjusted to 0.1%. Controls were also prepared with equivalent concentrations (0.1% *v*/*v*) of DMSO. For experiments, cultures were obtained by transplanting mycelium disks (10 mm diameters) from a single mother culture in the stationary phase. They were incubated at 26 ± 1 °C on SDA on thin sheets of cellophane until the logarithmic growth phase. Subsequently, the cultures were transferred to Petri plates with media containing 100 or 200 μg/mL of the single substance and incubated under growth conditions. The fungal growth was evaluated daily by measuring colony diameters (in millimeters) for seven days from the treatment onset.

The percent inhibition of growth was determined as the average of three different experiments.

IC_50_ values were obtained testing α-bisabolol and Fluconazole against the nine dermatophytes at different concentrations: 1, 5, 10, 20, 50 μg/mL for Fluconazole, 5, 10, 20, 50, 100 μg/mL for α-bisabolol.

IC_50_ values were determined as the average of three different experiments.

### 3.4. Determination of Optimal Spore Concentration-Growth in Sabouraud Dextrose Broth in Resazurin Assay

*M. gypseum* was grown on Sabouraud Dextrose (SD) agar at 28 °C until sporulation occurs, typically for 7–14 days. The spores were harvested in Sabouraud Dextrose (SD) agar from 12 days cultures and the numbers of Colony Forming Units (CFU) per mL were determined by plating serial dilutions on Sabouraud Dextrose agar plates.

Subsequently, serial dilutions were made from a first solution containing 6 × 10^6^ spores/mL in Sabouraud Dextrose Broth to obtain spore concentrations from 10^1^ to 10^6^ spores/mL. The vials were prepared in duplicates for each concentration. Then, 100 μL of a stock solution of Resazurin (Resazurin sodium salt, Sigma, stock solution of 0.00675 g/mL of sterile distilled water) was added to test vials. Vials were covered, gently rotated horizontally to mix the vial contents, and incubated in the dark at 24 °C. At six different incubation times (24, 48, 72, 96, 120 and 144 h) the percent reduction of Resazurin was determined by taking absorbance readings at two wavelengths, 540 and 630 nm. Duplicate negative control vials, contained 10 mL of medium and 100 μL of Resazurin only, were made.

### 3.5. Evaluation of Inhibition of Spore Germination of α-Bisabolol

The efficacy of α-bisabolol was evaluated by the Resazurin assay using optimized incubation time and spore density. A stock solution of α-bisabolol was prepared in DMSO. Then α-bisabolol was added to test vials in duplicate at the concentrations of 20 and 100 μg/mL. Test vials contained α-bisabolol, 10^5^ spore/mL (determined from previous evaluation), and 100 μL of Resazurin stock solution Sabouraud Dextrose Broth. The vials were covered, gently rotated horizontally to mix the content, and incubated in the dark at 24 °C for 120 h. Duplicate negative control vials contain 10 mL of medium and 100 μL of Resazurin stock solution. Duplicate positive control vials containe 10 mL of medium, 10^5^ spore/mL, and 100 μL of Resazurin stock solution.

### 3.6. Spectrophotometric Measurement and Visual Inspection

Absorbance data was expressed as percent Resazurin reduced as a function of incubation time. After 120 h, the percent reduction of Resazurin was determined colorimetrically by taking absorbance readings at two wavelengths, 540 and 630 nm, to compensate for an overlap in the absorption spectra of the oxidized and reduced forms of the dye [28]). Absorbance was read with a Beckman Coulter TM DU^®^ 530, Life Science UV/Vis Spectrophotometer Single cell module. The mean absorbance values were used in subsequent calculations. The following Equation (1) was used to calculate the percent reduction of Resazurin:

Percent reduction of Resazurin = [(O2 × A1) − (O1 × A2)]/[(R1 × N2) − (R2× N1)] × 100
(1)

where O1 = molar extinction coefficient (E) of oxidized Resazurin (Blue) at 540 nm, O2 = E of oxidized Resazurin at 630 nm, R1= E of reduced Resazurin (Red) at 540 nm, R2 = E of reduced Resazurin at 630 nm, A1 = absorbance of test vials at 540 nm, A2 = absorbance of test wells at 630 nm, N1 = absorbance of negative control vial (media plus Resazurin but no cells) at 540 nm, and N2 = absorbance of negative control vial (media plus Resazurin but no cells) at 630 nm. Molar extinction coefficients were 540 nm = 104,395, reduced and 47,619, oxidized; and 630 nm = 5494, reduced and 34,798, oxidized.

A blue color was interpreted as absence of metabolic activity (no spore germination). A fluorescent pink color was interpreted as presence of metabolic activity (spore germination). A purple color in the vials was interpreted as a trailing result where some metabolic activities were present but a longer incubation time allowed the purple color to change to pink.

### 3.7. Transmission Electron Microscopy (TEM)

For TEM, the youngest hyphae of *M. gypseum* were chosen from untreated mycelia and from mycelia treated for 24 h with 100 and 200 μg/mL of α-bisabolol.

The samples were fixed with 6% glutaraldehyde (GA) in a 0.1 M sodium cacodylate buffer, pH 6.8 for 6 h at 4 °C. After rinsing in the same buffer solution, they were post-fixed for 15 h with 1% osmium tetroxide (OsO_4_) in the same buffer, dehydrated in a graded series of ethanol solutions and embedded in Epon-Araldite resin. Sections were cut with an LKB Ultratome III, stained with uranyl acetate and lead citrate and observed under a Hitachi H-800 electron microscope at 100 kV (provided by the Electron Microscopy Center of Ferrara University).

## 4. Conclusions

In conclusion, we have shown that α-bisabolol, a natural product present in many preparations intended for cosmetic and dermatological uses, possesses significant antifungal activity *in vitro*, against dermatophytes. This property suggests its applicability, within a multidrug approach, for the treatment of dermatomycoses, which are especially persistent diseases in immunocompromised hosts [20]. Ongoing studies are now evaluating the synergistic combination of α-bisabolol with traditional antifungal agents for the treatment of dermatophytoses, and antifungal activity against other pathogenic fungi. Although many antifungals are available, their side effects and drug interactions, and the existence of resistant organisms have created a need to find safer and more effective treatments. Cytotoxicity assay will be performed to assess the selectivity of the compound in the same conditions as the antifungal test.
